# Maintaining Transient Diversity Is a General Principle for Improving Collective Problem Solving

**DOI:** 10.1177/17456916231180100

**Published:** 2023-06-27

**Authors:** Paul E. Smaldino, Cody Moser, Alejandro Pérez Velilla, Mikkel Werling

**Affiliations:** 1 Department of Cognitive & Information Sciences, University of California, Merced; 2 Santa Fe Institute, Santa Fe, New Mexico; 3 Interacting Minds Centre, Aarhus University

**Keywords:** allied field: computer science, allied field: sociology, cognition, collective intelligence, culture/diversity, diversity, formal models, intragroup processes, networks, social cognition

## Abstract

Humans regularly solve complex problems in cooperative teams. A wide range of mechanisms have been identified that improve the quality of solutions achieved by those teams on reaching consensus. We argue that many of these mechanisms work via increasing the *transient diversity* of solutions while the group attempts to reach a consensus. These mechanisms can operate at the level of individual psychology (e.g., behavioral inertia), interpersonal communication (e.g., transmission noise), or group structure (e.g., sparse social networks). Transient diversity can be increased by widening the search space of possible solutions or by slowing the diffusion of information and delaying consensus. All of these mechanisms increase the quality of the solution at the cost of increased time to reach it. We review specific mechanisms that facilitate transient diversity and synthesize evidence from both empirical studies and diverse formal models—including multiarmed bandits, NK landscapes, cumulative-innovation models, and evolutionary-transmission models. Apparent exceptions to this principle occur primarily when problems are sufficiently simple that they can be solved by mere trial and error or when the incentives of team members are insufficiently aligned. This work has implications for our understanding of collective intelligence, problem solving, innovation, and cumulative cultural evolution.

Humans and other social animals often solve problems in teams or collectives. They forage for food or nesting sites. They explore technological designs. They deliberate over evidence to make decisions. Case studies, behavioral experiments, and formal models have been used to identify a wide variety of mechanisms that allow teams to reach higher-quality solutions. Zollman (2010) first introduced the notion of *transient diversity* (though see also Grassle & Sanders, 1973; Lyback, 2003) in the context of collective problem solving in teams. The idea is that a successful team will maintain a diverse set of solutions so that good solutions are not unexplored. But importantly, this diversity of solutions should be *transient* so that the diversity does not persist long enough to hinder convergence to a common solution. We argue here that evidence from across several different modeling paradigms indicates a far more general principle for collective problem solving than previously indicated: *Any* mechanism that extends the transient diversity of solutions in the population will improve the quality of the solution on which the group ultimately converges.

To be clear, our claim is not that transient diversity is the only mechanism that improves collective problem solving, nor do we here provide an exhaustive list of all instances in which transient diversity improves collective problem solving. Further, there are some minor caveats to the general claim, which we will explore in detail. Over the years, many mechanisms have been discussed for improving the solution quality of problem-solving teams. Our central argument is that many (if not most) of these mechanisms are best viewed as different means of increasing the transient diversity of the population.

*Diversity* is a term with many possible meanings. We focus on the diversity of solutions to a well-specified problem. That is, the diversity of a population is an instantaneous measure of the variation of solutions under consideration. Why does transient diversity lead to higher-quality solutions? When a wider area of the solution space is explored, the population becomes more likely to find an optimal or high-quality solution and less likely to become stuck on a local optimum. The longer that diversity persists, the larger the total area of solution space being explored becomes. Rapid consensus can be important when decisions must be made quickly, but consensus also precludes certain questions from being asked and certain ideas from being explored. This highlights the important trade-off between speed and accuracy in problem solving (Grim et al., 2013). Increasing transient diversity means that a solution is more likely to be of higher quality, but it also increases the time it takes for a team to reach consensus. A similar phenomenon is well known for cases of individual-level problem solving (Hourihan & Benjamin, 2010; Raviv et al., 2022; Vul & Pashler, 2008). The overall value of transient diversity therefore depends on the relative importance of solution quality versus timely decision-making. In this article, we focus on solution quality alone, but this trade-off should be kept in mind.

There are numerous mechanisms that can produce more diverse populations or maintain high levels of diversity for longer times. These are often studied in isolation as separable mechanisms that improve the solutions discovered by cooperative teams. Our proposal is that these are better appreciated as mechanisms for increasing transient diversity. We can draw an analogy from research on social evolution. Over several decades, researchers identified numerous mechanisms to facilitate the evolution or maintenance of altruistic cooperation. These include kin selection, direct and indirect reciprocity, group structure, limited dispersal, and partner choice. All of these mechanisms are now understood to be different ways of generating *positive assortment*, which means that interactions occur between individuals using the same behavioral strategies at rates higher than predicted by chance (Apicella & Silk, 2019; Fletcher & Doebeli, 2009; Nowak, 2006), allowing the benefits of cooperation to be preferentially bestowed on cooperative individuals. We propose that transient diversity operates similarly as a unifying principle for improving the quality of collective problem solving.

We anchor our review on findings from formal modeling studies. There are quite a few different modeling paradigms used to study collective problem solving, but if we can show that they all converge on similar results, that would indicate the presence of a general principle for systems with the properties shared among the different models. Below, we first review the types of models we use as evidence, which originate from a wide range of disciplines. We then enumerate specific mechanisms for generating transient diversity and explore how each does so. We then briefly review some of the relevant empirical work and discuss the extent to which model assumptions are met in these studies. Finally, we discuss limitations to our proposal.

## Models of Collective Problem Solving

The models we consider have several core assumptions in common. First, the problem being solved is assumed to be well specified, such that solutions can be directly compared and assessed for quality. Second, the problem is assumed to be sufficiently complex so that individuals are unlikely to find the best solution on their own. Third, each individual is assumed to prefer to adopt the best solution they know of, and individuals will agree on the quality of a particular solution. And fourth, teams are assumed to be cooperative, such that individuals’ goals are aligned, and they willingly share information with others. Even constrained by these assumptions, there are several ways a system of collective problem solvers can be formalized. Below, we briefly describe some of the best known and widely used of these models (see Table 1).

**Table 1.** Summary of Models Considered

Model	Key characteristics	Key references
NK landscapes	Frames the search for solutions to a problem as travelling through a landscape and finding the highest point (the optimum) on that landscape	Barkoczi & Galesic (2016); Gomez & Lazer (2019); Lazer & Friedman (2007)
Hong-Page model	Conceptualizes the problem-solving system as a collection of individual problem solvers with different perspectives (initial beliefs) and heuristics (solution-generating algorithms)	Hong & Page (2001, 2004)
Organizational-learning models	Model the feedback individuals have on a larger organization and the subsequent feedback organizations have on individuals	Fang et al. (2010); March (1991); Miller et al. (2006)
Network-epistemology models	Model the quest for the best solution to a problem as a choice between different slot machines (bandit problems) in a social context	Bala & Goyal (1998); Kummerfeld & Zollman (2016); Zollman (2007, 2010)
Potions model	Conceptualizes generating new solutions to a problem as synergizing previous innovations to cumulatively reach better solutions	Derex & Boyd (2016); Migliano et al. (2020); Moser & Smaldino (2023)
Evolutionary models	Model the fixation of adaptations and the discovery of novelty by populations of replicators in a fitness context	Boyd & Richerson (1985); Taylor & Jonker (1978); Wright (1931)

We purposefully separate the descriptions of the models from the results derived from those models, both because each model is associated with multiple results and because many of those results are shared between models. However, we want to highlight that each of these models entails two forms of representation: those of the collective problem tasks to be solved and those of the cognitive and behavioral processes that agents and teams employ to solve those tasks. As with many models of social phenomena (Smaldino, 2023), these latter representations tend to be fairly minimal, employing simple “greedy search” and “copy the best” heuristics in order to focus on emergent collective patterns. Such simple heuristics are likely to be adaptive when faced with a diverse set of problems and may be reasonable approximations of human behavior at scale (Gigerenzer & Brighton, 2009).

### NK landscapes

The NK landscape model was first formulated to characterize epistasis in gene regulatory networks (Kauffman & Levin, 1987; for a primer, see Csaszar, 2018). Social scientists have used the NK landscape as a model of problem solving (Lazer & Friedman, 2007), where each of *N* bits represents the presence or absence of some solution element, and the parameter *K* represents the number of interdependencies between those elements. Landscapes where *K* is close to zero can be solved by hill climbing and are viewed as simple problems, whereas *K* close to *N*/2 characterizes complex problems where hill climbers get stuck on local optima. Models typically assume a networked team, each starting with a unique solution and searching individually via hill climbing while also sharing information with neighbors. Lazer and Friedman (2007) showed that although simple problems were solved most effectively on dense networks, more complex problems were best tackled by sparsely connected teams. A strength of this model is that the complexity of the problem and the size of the solution space can be easily manipulated.

### The Hong-Page model

Hong and Page (2001, 2004) considered a model in which, like the NK landscape model, solutions are represented as bit strings of *n* points, and the value of each bit string is derived from random mapping. Their model focuses on individual differences regarding the heuristics each agent uses to search the solution space. Solutions are mapped onto adjacent points on a circle. Agents attempt to find maximum values by searching clockwise among the *n* points using individual heuristics. This allows them to check *k* positions that lie clockwise from the group’s agreed-on maximum point, thereby moving around the circle and testing possible solutions. At each time step, agents start with the previous agent’s best solution. Solutions are translated to individual cognitive representations, and individuals’ search heuristics can vary. In this way, some agents are more likely than others to find high-quality solutions when searching alone, which explicitly affords a comparison of group-level diversity with individual-level talent. Hong and Page (2001, 2004) demonstrated that diverse teams with only midlevel individual talent could outperform more homogenous teams of individual top performers.

### Organizational-learning models

March’s (1991) model of organizational learning examines the behavior of firms where individual agents are tasked with learning about an external reality comprised of *m* dimensions, each with a binary value of 1 or −1. Individuals hold beliefs about the value of each dimension of reality as either 1, –1, or 0. In turn, the organization similarly holds “beliefs,” which represent the collective beliefs of the firm. Individuals’ beliefs can be changed to the beliefs of the firm, and the beliefs of the firm can be altered by the level of agreement within the firm, with the rates of change for both individual- and organization-level beliefs controlled by independent parameters. March (1991) found that organizations performed better when individuals were slower to become socialized to the beliefs of the collective. As this model involves both the influence of individuals on an organization and the influence of the organization on those individuals, it has been useful for understanding ideal arrangements of individual contributors to collective problems.

### Network-epistemology models

A standard benchmark problem in machine learning is the multiarmed bandit where each “arm” yields payoffs drawn from a unique distribution function. The learner’s goal is to maximize its cumulative payoff by consistently choosing the best option with the least amount of exploration. Researchers interested in social learning have considered networked populations of learners who can explore solutions individually but also learn about payoffs from observing the consequences of others’ actions where the focus is typically on the value of social learning (Bala & Goyal, 1998; Rendell et al., 2010; Turner et al., 2023). Individuals can update their estimates of each arm’s payoff through Bayesian learning. Using these models as starting points, philosophers interested in social epistemology and the sociology of science have found that larger, more sparsely connected networks are more likely to converge on the correct belief in the form of the higher-paying bandit arm (Zollman, 2007). A strength of this model is that it incorporates uncertainty and the idea that individual observations can be misleading.

### The potions model

The models described above assume that the ability to adopt a solution is independent of prior history, such that any individual could adopt any solution. However, many technologies and behaviors are possible only with specific prior knowledge. This idea was formalized in the potions model (Derex & Boyd, 2016), first as a multiplayer game using human participants and later as an agent-based model (Cantor et al., 2021; Migliano et al., 2020; Moser & Smaldino, 2023). Each agent begins with a set of ingredients that can be combined into a potion to stop the spread of a harmful virus. The efficacy of the potion depends on the ingredients used. Critically, once an efficacious recipe is found, it can then serve as a stand-alone ingredient to be combined with two other ingredients for a future potion. These innovations can accumulate iteratively, resulting in potions of ever-increasing efficacy. Agents explore combinations of ingredients through individual trial and error but can also learn from neighbors. Derex and Boyd (2016) showed that a population divided into small groups that interacted only intermittently could outperform a population in which individuals could access everyone else’s information simultaneously. Not only does this model allow for the consideration of cumulative innovation, but it also affords the consideration of path dependency at the population level, as different subgroups may discover different cumulative solutions that can then be further combined when the subgroups interact and learn from one another.

### Evolutionary models

The paradigmatic model of evolution by natural selection, commonly known as the replicator dynamic, explicitly links the variance in strategies within a population with the intensity of selective pressure the population experiences (Schuster & Sigmund, 1983; Taylor & Jonker, 1978). Early perspectives on the fixation of beneficial alleles and novelty in populations and the optimization problem were typified in theories of Sewall Wright (Wade & Goodnight, 1998; Wright, 1948). As the originator of the “adaptive landscape” analogy, now commonly used in machine learning and computational models of collective intelligence, Wright saw populations as inhabiting landscapes of optimal and suboptimal adaptations (Wright, 1931). More successful strategies generate more replicates of themselves relative to less successful strategies, leading to a decrease in strategy diversity as selection acts on the population. In this way, a population can converge on local optima (the “peaks”) of the fitness landscape. However, finding a global optimum will often not be as straightforward a task because the environmental features and the space of possible strategies can translate into rugged, difficult-to-traverse fitness landscapes (Gavrilets, 2004). By exploiting mechanisms that generate diversity (such as random mutations in individual strategies), populations can get nudged beyond local peaks onto potentially more advantageous ones. Similar arguments have also been formalized for human cultural evolution (Boyd & Richerson, 1985).

## Mechanisms for Increasing Transient Diversity

There are a wide range of mechanisms that produce transient diversity. Here, we review several of them, providing evidence from across modeling frameworks and explaining how each mechanism leads to transient diversity (see Table 2). This is not meant to be a complete or definitive list. Indeed, it is likely that there are mechanisms that fit the bill that have yet to be identified. Instead, we use it to illustrate how the unifying characteristic among seemingly disparate mechanisms is that they all work by increasing transient diversity.

**Table 2.** Summary of Mechanisms That Promote Transient Diversity

Mechanism	Description	Key references
Diversity of initial beliefs	Diversity is maintained by simply having agents with different starting points.	Boroomand & Smaldino (2021); Clarke (1979); Gomez & Lazer (2019); Walter et al. (2018); Zollman (2010)
Diversity of search strategies	By having a diverse set of individual search strategies, more of the search space can be explored.	Boroomand & Smaldino (2021); Gomez & Lazer (2019); Hong & Page (2001, 2004); March (1991); McElreath et al. (2013); Kummerfeld & Zollman (2016)
Sparse networks	Sparser networks (i.e., those with lower average degree and/or longer average path length) slow the diffusion of information compared with more connected networks. This gives distant parts of the network time to explore different parts of the solution space without assimilating each other’s solutions.	Cantor et al. (2021); Derex & Boyd (2016); Fang et al. (2010); Lazer & Friedman (2007); Migliano et al. (2020); Moser & Smaldino (2023); Wright (1931, 1948, 1982); Zollman (2007, 2010)
Slow or intermittent interactions	When communication rates between individuals are reduced, social learning is attenuated. This leads to a decrease in how much solutions are alike between neighbors, which helps maintain higher levels of solution diversity.	Derex & Boyd (2016); March (1991); Migliano et al. (2020); Moser & Smaldino (2023)
Communication noise	Imperfect copying of candidate solutions makes perfect conforming less likely and results in more variance of solutions.	Boroomand & Smaldino (2022)
Behavioral inertia	Reluctance to adopt the solutions of others, except if they are substantially better than one’s own, keeps potentially beneficial pathways of innovation open for longer.	Boroomand & Smaldino (2022); Gabriel & O’Connor (2022); Walker et al. (2021)
Context biases for social learning	Preferentially learning from certain people or aggregating multiple sources of information can decrease the proclivity to adopt marginally better solutions, prolonging the maintenance of varied suboptimal strategies.	Barkoczi & Galesic (2016); Fazelpour & Steel (2022); Kendal et al. (2018)
Outgroup distrust	By distrusting the information given from an outgroup, a group of members can maintain their current beliefs for longer periods of time.	Fazelpour & Steel (2022); Wu (2022)

### Higher variance of initial solutions

One way to increase transient diversity is to simply begin with a greater diversity of initial solutions. Another way is through the use of larger teams, which are likely to involve more perspectives through sheer force of numbers. Using an NK landscape model, Boroomand and Smaldino (2021) showed that increasing both the diversity of initial solutions and the overall team size improve solution quality for complex problems. Gomez and Lazer (2019) further showed that this type of diversity works best when like-minded people are placed in connected subnetworks, which allows for local consensus but maintains the overall diversity of the network for longer. Zollman (2010), using a network-epistemology approach, showed that populations with wider (more uncertain) priors were more likely to reach consensus on the correct solution. In evolutionary biology, the evolution of more robust polymorphisms can be facilitated through processes that increase diversity of phenotypes, including mutation, frequency dependence, and the colonization of and migration among heterogeneous environments (Clarke, 1979; Walter et al., 2018). In economics, Wärneryd (2002) showed that in a population of risk-sensitive rational actors performing in winner-takes-all types of situations, a broad initial distribution of risk preferences (in which diversity is maximized) leads to efficient rent dissipation as the system evolves to a distribution where all attitudes to risk are represented.

### Greater diversity of individual search strategies

Another way to maintain diversity is to have people search in different directions. Having a diversity of strategies (also referred to as diversity of abilities or heuristics) means that individuals can explore a wider area of solution space. In March’s (1991) organizational-learning model, introducing heterogeneity to individuals’ learning rates allowed organizations to produce more accurate models of reality than organizations composed exclusively of fast- or slow-learning individuals. Hong and Page (2001, 2004) showed that a team of problem solvers who were diverse in the way they explored the solution space could outperform a less diverse team, even when the members of the latter team reached higher-quality solutions when searching individually. Gomez and Lazer (2019) found similar results using an NK landscape model, showing that a diversity of search strategies led to higher-quality solutions and, moreover, that teams performed better when individuals with diverse abilities were intermixed within network clusters, as this could counteract local consensus achieved through sharing information. Boroomand and Smaldino (2021), also using an NK landscape model, showed that the presence of risk-taking agents, who simultaneously varied multiple solution elements chosen at random, also increased solution quality by increasing transient diversity. Kummerfeld and Zollman (2016), using a network-epistemology approach, similarly showed that moderate amounts of random exploration on the parts of individuals could improve the likelihood that the group reached consensus on the correct solution. In cultural evolution, models have shown that the introduction of diversity through migration makes directional social learning biases adaptive with respect to individual and unbiased social learning (McElreath et al., 2013), unless migration is so strong that diversity itself is stifled. When extended to environmental stochasticity in time, these models show that strategies that combine individual and social learning can lead to a tangible evolutionary advantage over pure strategies, as they spread risk out across the learning substrategies.

### Sparse networks

It is a well-known result that information spreads more rapidly on densely connected networks and on networks with short average path lengths (Lind & Hermann, 2007). If a problem is sufficiently simple that a single individual can quickly find a solution and the key concern is therefore communicating that solution throughout the network, then dense networks are best (Centola, 2022; Lazer & Friedman, 2007). However, when problems are complex, such that individual search is likely to become stuck on a local optimum, rapid consensus becomes less desirable. Sparser networks (i.e., those with lower average degree and/or longer average path length) facilitate slower percolation of information, which leaves time for disparate regions of the network to explore different regions of solution space. Early relevance of this idea to the search problem in population genetics was recognized by Wright. In his shifting-balance theory, Wright (1948) proposed that a global population split into subpopulations with limited gene flow between them allows for the global population to explore nonoptimal areas of solution space and therefore discover otherwise inaccessible novelty. This influence of network sparsity on solution quality has been similarly observed using NK landscape models (Lazer & Friedman, 2007), organizational-learning models (Fang et al., 2010), network-epistemology models (Zollman, 2007, 2010), and the potions model (Cantor et al., 2021; Migliano et al., 2020; Moser & Smaldino, 2023).

### Slow or intermittent interactions

Network models are often based on the assumption that connections are fixed and that transmission between connected nodes is deterministic. However, solution quality can be improved if the communication or diffusion rate is decreased so that learning from neighbors becomes probabilistic. This is analogous to reducing the transmissibility of a contagion in an epidemiological model. Reducing the communication rate means that individuals do more individual search and less social learning, which decreases the correlation in solutions among neighbors and thereby helps maintain higher levels of solution diversity in the population. In his seminal article on organizational learning, March (1991) altered learning rates between individuals and their organization, finding that slower learning rates led to better organizational performance, albeit at a loss to time until the organization obtained consensus. A similar way to achieve such diversity is to isolate different groups or network clusters and allow them to connect with other clusters only intermittently. Separation affords each cluster path independency, allowing each to converge on a distinct solution. This is particularly important in models that allow for the cumulative recombination of solutions, in which the combination of two solutions can be valued more highly than either solution in isolation (Derex & Boyd, 2016; Migliano et al., 2020; Moser & Smaldino, 2023).

### Communication noise

Models very often assume that communication is perfect and do not account for errors of either transmission or perception. However, diversity can be maintained purely by accident if a solution is copied with error. Boroomand and Smaldino (2022) studied an NK landscape model in which, during social learning, each element of a target solution was correctly learned with probability 1 – *c*, and otherwise the learner substituted the solution element from their current solution. Though framed as noise, this could be viewed in other ways, including as a form of strategic selective copying. Either way, it was found that increasing noise levels (up to 50% considered) improved the overall solution quality by maintaining a higher diversity of solutions. This result is similar to the verbal predictions made by Eisenberg (1984) and other scholars suggesting an adaptive role for ambiguity in communication.

### Behavioral inertia

Individuals can sometimes be stubborn, favoring their own ideas over the ideas of others even when their own ideas are inferior. Pragmatically, it may also be costly to abandon one’s own “good enough” solution for someone else’s solution that is only slightly better, yielding a negative net benefit. Either of these factors can lead to a form of behavioral inertia in which another solution must be substantially better than one’s current solution to justify its adoption. Interestingly, this reluctance to learn socially can maintain a diversity of solutions and keep potential pathways in solution space open for longer. In other words, behavioral inertia can increase transient diversity. This effect has been demonstrated to improve solution quality in both NK landscape models (Boroomand & Smaldino, 2022) and network-epistemology models (Gabriel & O’Connor, 2022). Similarly, in a model of cultural innovation in which agents could either copy the strategies of others or innovate their own strategies, Walker et al. (2021) found that the introduction of a third strategy, “maintain,” led to stronger population-level adaptation than only copying or innovating standing strategies.

### Context biases for social learning

Conditions for learning that impede efficient information flow appear to prolong transient diversity. Another mechanism that produces this impediment involves nonrandom social learning strategies. In particular, *context-dependent biases* are proclivities to learn preferentially from certain people or to aggregate multiple sources of information in nonuniform ways (Kendal et al., 2018). Barkoczi and Galesic (2016) studied an NK landscape model in which agents sampled a subset of their neighbors and employed either success-biased or conformist social learning strategies. Although all of the considered strategies outperformed pure individual learning, the strategy that produced the best solutions to complex problems was conformist transmission with small samples. The conformist rule reverted to individual learning when there was no majority among the sampled solutions, and so copying occurred only when multiple agents converged on the same high-quality solution. This allowed the diversity of solutions to be maintained for long enough that high-quality solutions were vetted by receiving “votes” from multiple searchers. Note that a conformist learning bias differs from social pressures to conform, which are likely to reduce diversity. And indeed, using a network-epistemology model, Fazelpour and Steel (2022) showed that pressure to conform to the solution of one’s neighbors reduces the probability that the group reaches consensus on a high-quality solution.

### Outgroup distrust

In addition to holding individual-level biases for self versus other, as with behavioral inertia, individuals can also exhibit group-level biases. If members of one group give less weight to information from outgroup individuals, they maintain their current beliefs for longer. If this bias is very strong, individuals ignore useful information, which can lead to consensus on poor solutions or even polarization (O’Connor & Weatherall, 2018). However, a small amount of outgroup distrust might serve merely to prolong transient diversity and by doing so improve group-level outcomes. Using a network-epistemology approach with a two-group population, Fazelpour and Steel (2022) demonstrated that small levels of distrust for information from outgroup members improves the probability of consensus on high-quality solutions. Complicating this matter, Wu (2022) considered a similar two-group network-epistemology model, in which a dominant group completely ignored information from the marginalized group, whereas the latter group used information from the former. Because the dominant group makes updates on less evidence, it maintains its diversity of belief for longer. However, it is the marginalized group that benefits from this exploration. Compared with a similar population without any group biases, the marginalized group was more likely to converge on the correct belief, whereas the dominant group was less likely. This indicates that groups may not always be the ones to benefit from their own diversity when informational asymmetries exist.

## Empirical Evidence

The contribution of transient diversity to group success has long been recognized in studies of organizational behavior and group psychology. Bavelas (1950) showed a relationship between more fragmented group structures and task efficiency. This relationship was further explored experimentally by Guetzkow and Simon (1955), who placed individuals into groups of five and gave them each a card with a set of symbols; the task was to find the symbol they all had in common. They found that organizational arrangements with the fewest open communication channels performed best.

Other empirical studies of collective problem solving have shown mixed support for the benefits of transient diversity. One challenge is that it is often difficult to disentangle factors that lead to greater diversity of solutions from other types of diversity, including those that may reduce the alignment of incentives and goals. In a simulated caucus task, in which university students were asked to elect an ideal candidate from an applicant pool, initial distributions of diverse unshared information about candidates did not alter who was actually elected, as most people discussed only the information that others in the group had already shared (Stasser & Titus, 1985). This *shared-information bias* has also been found in other empirical studies of collective decision processes (Stasser & Stewart, 1992; Wittenbaum, 1998).

In a meta-analysis, Joshi and Roh (2009) examined the role that both relations-oriented (e.g., gender, race) and task-oriented (function, education, tenure) diversities played in group performance. Across all studies, task-oriented diversity related to function (i.e., specialization within an organization) was the only consistent positive predictor of team performance. The effect of other forms of diversity was typically either negative or virtually zero. However, when individuals had only a short amount of time to complete their tasks, relations-oriented diversity also had a positive effect on performance. In this case, the time limits may have effectively prevented convergence on shared solutions, similar to the role that *transient* diversity of solutions plays in the models described above.

The relationship between diversity and real-world performance may also be nonmonotonic. Aggarwal et al. (2019) studied the diversity of cognitive styles in an experimental game in which individuals in small groups were prevented from communicating directly but had to choose from a set of options; the group’s payoff depended on the choices of each member. Teams with an intermediate level of diversity outperformed teams with both low and high levels of diversity (teams with the highest levels of diversity performed worst, perhaps because of an inability to coordinate in the absence of communication). These empirical results resemble findings from models (Derex et al., 2018; Fang et al., 2010), in which intermediate levels of network connectivity are ideal for group performance; similar concepts have been framed as “optimum mutation rate[s]” in population biology (Crow, 1986, p. 520).

Empirical studies have also tested the effect of specific network structures on the ability of human groups to solve tasks. Mason et al. (2008) found that in problems with only one solution, fully connected networks outcompeted small-world networks. When the landscape was more complex with more local solutions, small-world networks outperformed fully connected networks. Similar results have been obtained with human participants in the potions game, in which no fully connected groups were able to overcome the inherent path dependencies in the game, whereas over half of the partially connected groups were able to do so (Derex & Boyd, 2016). Nonetheless, a large-scale study by Mason and Watts (2012) failed to find a positive role for partial connectivity, finding instead that fully connected networks performed best in complex tasks with local maxima. Although this might appear to be a strike against the transient-diversity hypothesis, we should consider the behavior of the agents themselves: Unlike the agents in agent-based models, humans in fully connected groups maintained a diversity of strategies despite their connectedness. To explore this phenomenon further, Shore et al. (2015) studied a similar task on networks and carefully considered cognition. They found that human players possessed an awareness of what their neighbors were doing; instead of copying their neighbors, they employed a strategy of limiting wasteful redundant exploration of the solutions that were known. Thus, human cognition may maintain diversity when network structure fails to do so.

## Limitations

Although we have argued that transient diversity is an important factor in a wide variety of collective-problem-solving frameworks, it is also necessary to explore situations in which it may fall short of its proposed virtues; some of these are illustrated in the empirical examples above. In scenarios where rapid consensus is important, marginal increases in solution quality must be weighed against the costs of further exploration. Similarly, in simpler or smoother landscapes of possible solutions, dense or flat network structures tend to outperform more structured networks (Lazer & Friedman, 2007; Shore et al., 2015). Such landscapes represent problems in which there are few interdependencies between problem components, reducing the problem to a set of dials that can be tuned independently (Simon, 1962)—this sort of problem can be easily solved through individual learning, and thus the solution needs only to be broadcast by the first team member to find it.

Further, the mechanisms we listed may not contribute additively to solution quality. For example, several studies have indicated that efficient rather than sparse networks are preferable when other mechanisms for maintaining transient diversity are present (Barkoczi & Galesic, 2016; Foley & Riedl, 2015; Zollman, 2010). The value of transient diversity also relies on the assumption that the interests of team members are aligned. However, diversity of interests can erode cooperation. For example, O’Connor and Weatherall (2018) examined a network-epistemology model in which agents devalue information from those with differing beliefs. They found that this can lead to polarization, in which a large proportion of the population holds an incorrect belief. Transient diversity may lead to harm when the benefits of rapid consensus outweigh the benefits of improved solutions (Wu & O’Connor, 2023). Relatedly, we have not considered scenarios such as optimal behavior during collective behaviors (e.g., voting), in which diversity may translate to an inability to reach consensus. Moreover, consensus usually implies that the collective will take action—teams do things when they find a solution. These actions may create new opportunities for further collective problem solving, but they can also create conditions in which previously adaptive strategies no longer hold. The implications of this sort of continuous collective adaptation are discussed in Galesic et al. (2023).

The models we reviewed make several assumptions that constrain our ability to uncritically generalize from them. First, they assume that problems are well specified, so that solutions can be directly compared. In reality, most solutions involve trade-offs between several short- and long-term costs and benefits, at least some of which can only be guessed at. This can make direct comparisons difficult. Second, the models focus on complex problems that cannot easily be solved by individual learning. As noted, simple problems tend to benefit more from raw computing power than from diversity. Third, the models assume that individuals have sufficiently aligned knowledge and perception that they can readily agree on a rank ordering of solutions. Although a variety of perspectives may be a path to useful transient diversity, it can also lead to disagreement and stalemate. And fourth, the models assume that team members have aligned incentives and cooperate accordingly. In reality, even in cooperative groups, individuals may have divergent incentives and may belong simultaneously to multiple groups with competing interests. Each community can be viewed as “an ecology of games” (Long, 1958), where rational behavior within a group may be irrational when one considers how outcomes are summed across the groups in which an individual participates.

## Conclusion

Given the convergence from multiple models and mechanisms for maintaining it, the use of transient diversity by groups of agents to improve solutions likely represents a general principle for collective problem solving. The multiple mechanisms that can be used to create and maintain it can be shown to transfer across a number of models and a diversity of tasks. A critical consideration for the use of transient diversity to leverage group abilities is the ideal level of diversity that should be maintained within a population. With too little diversity, populations may rapidly and prematurely converge on nonideal solutions, leading to deadlocks, polarization, and path-dependent lock-in at local optima. With too much diversity, consensus may be difficult to obtain and, in situations that require speedy solutions, may simply be too costly. An important assumption of most studies reviewed here is a *lack* of diversity in agent goals, such that interests were always perfectly aligned. The interaction of multiple types of diversity is an important consideration for future research on collective problem solving.

## Transparency

*Action Editor:* Henrik Olsson

*Editor:* Interim Editorial Panel
